# Acknowledging a Global South phenological science: the use of phenological and meteorological indicators in indigenous knowledge systems in Rural South Africa

**DOI:** 10.1007/s00484-026-03201-9

**Published:** 2026-04-16

**Authors:** Sinoxolo Magaya, Jennifer M. Fitchett

**Affiliations:** School of Geography, Archaeology and Environmental Studies, University of the Witwatersrand, Johannesburg, South Africa

**Keywords:** Phenology, Indigenous knowledge systems, Subsistence agriculture, Climate, South Africa

## Abstract

The majority of published phenological research relies on what is perceived to be scientifically robust sources of data – ground-based observations, webcams and remote sensing. This has resulted in a body pf phenological literature which remains skewed to the Global North. In much of the Global South, phenological events have been a key component of inter-generational Indigenous Knowledge Systems surrounding the daily and seasonal weather forecasting for small-scale agriculture. Under climate change, this critical awareness of the natural environment and the use of phenology to mark seasonal change can lead to a more rapid adaptation to changes missed by meteorological forecasting in the short term, while local extirpation of key indicator species limits long-term efficacy. This study explores these issues in the context of two small indigenous rural communities in South Africa who are heavily reliant on rainfed subsistence agriculture. Across both communities, observations of phenological events, weather and astronomical conditions are used by farmers to inform the timing of sowing, harvesting, and the selection of crops. However, the disappearance of key indicator species is threatening this approach, with the community raising concerns about the anthropogenic influence. These results demonstrate the critical value of indigenous knowledge as part of the phenological discourse, particularly in the face of global change.

## Introduction

Early published phenological research was very strongly concentrated in the global North, with key focus regions across Europe, the United Kingdom and North America (Parmesan 2007; Fitchett et al. 2015). The most recent systematic review on phenological research, conducted by Hassan et al. (2024), indicates that this pattern has not shifted over the most recent decade. This is in part due to an under-representation of work published in languages other than English, and a limitation on search terms, which results in regions such as Brazil – which are vibrant hubs of phenological research – being under-counted (Morellato et al. 2025). A bigger issue is that much of the published literature, and many of the reviews thereof, focus on what are deemed to be scientifically robust sources of phenological data – ground-based observations often within botanical and sometimes specifically phenological gardens (e.g. Nordt et al. 2021; Primack et al. 2021), remote sensing (e.g. Dronova and Taddeo 2022; Rahimi and Chuleui 2025) and webcams (e.g. Alberton et al. 2017; Jose et al. 2023; John et al. 2024). The phenological literature, therefore, largely neglects the more intrinsic observation of phenological events within rural communities to inform their agricultural practises, which remain commonplace particularly in the Global South.

The use of phenological indicators to signify seasonal change and predict weather conditions predates both the body of research on phenology and the availability of national weather service forecasts in many African countries (Jiri et al. 2016; Orlove et al. 2010), and indeed is often carried down over much longer inter-generational periods through oral histories. Rural agricultural communities frequently pay attention to the natural environment for significant cues to inform their farming and domestic activities (Fitchett and Ebhuoma 2018). While urban communities similarly do take note of environmental changes, as indicated in the case of the shifting flowering dates of Jacaranda trees in the Gauteng-City Region of South Africa (Fitchett and Raik 2021), in rural communities this is often more deliberate and consistent. Indigenous or Traditional Phenological Knowledge (TPK) is informed by ground-based phenological observations that depend on predictable sequences of environmental functions such as the response of plants and animals to climate variables and intra-seasonal variability (Armatas et al. 2016; Zuma-Netshiukhwi et al. 2013; Bastian and Bayliss Hawitt 2023; Clapcott et al. 2025). However, where these same observations are considered to be valuable datasets in the Global North when captured in naturalists’ diaries (e.g. Sparks and Carey 1995; Primack et al. 2023), these less physically tangible oral histories and narratives are often overlooked (Chambers et al. 2017; Fitchett and Ebhuoma 2018). Drawing from Traditional Ecological Knowledge (TEK) or Indigenous Knowledge Systems (IKS), mainly in the form of phenological indicators is one of the approaches that is gaining traction in research that aims to contribute to the reduction of environmental change risks in many rural areas (Radeny et al. 2019). Phenological indicators are significant in agricultural management because they indicate impacts of environmental change at a relatively high spatio-temporal resolution, such as that of individual villages and farming regions (Fitchett and Ebhuoma 2018). Furthermore, the predictable sequence within which the timing of plants and animals’ life cycle events occurs promotes effective development of pragmatic mitigation and adaptation measures for most indigenous rural and subsistence agriculture-dependent communities (Armatas et al. 2016; Chambers et al. 2013). This is important because it has relatively low input requirements which accommodate the socio-economic conditions of most indigenous rural and subsistence farmers (Bakre and Dorasamy 2015).

Subsistence agriculture in developing countries is argued to be the most vulnerable sector to the impacts of climate change (Seaman et al. 2014). However, several studies (Chand et al. 2014; Ebhuoma and Simatele 2017; Gadzirayi et al. 2006; Kijazi et al. 2013; Orlove et al. 2010) have demonstrated that rural financially-strained communities are not passive actors. Rather, they have comprehensive knowledge systems that ensure preparedness and effective responses to ecological changes and challenges over time (Plotz et al. 2017). In many African countries, phenology based indigenous knowledge (IK) is used to forecast and prepare for precipitation or periods drought, reducing the impacts of these climatic events, because agriculture is dominantly rain-fed (Jiri et al. 2016; Orlove et al. 2010). Subsistence farmers in rural areas use phenological and meteorological indicators to forecast daily and seasonal weather conditions (Chambers et al. 2017), which in turn displays the ability of rural subsistence agricultural communities to adapt to the changing environment and sustain their livelihoods (Ebhuoma and Simatele 2017). The literature similarly captures that rural agricultural communities have a repertoire of phenological knowledge that they have been observing and using to ensure food production while limiting the risks associated with climate change (Kijazi et al. 2013). However, indigenous farmers’ dependence on plant and animal life cycle events for seasonal and daily weather forecasting means that the projected shifts in phenology due to climate lead to an uncertain future (Chambers et al. 2013, 2017).

The present study explores the awareness of phenology, and the use of TEK and IKS in forecasting daily and seasonal weather in the indigenous subsistence agricultural communities of Umzimkulu and Lusikisiki, located across two of South Africa’s Provinces. Our study further explores concerns among the community regarding the impact of climate change on their continued reliance of these phenological indicators. Through exploring and documenting the role of phenology in the weather and climate adaptations employed by these indigenous rural communities, we present the distinct value of phenological knowledge in the global South.

## Study Site

**Fig. 1:**
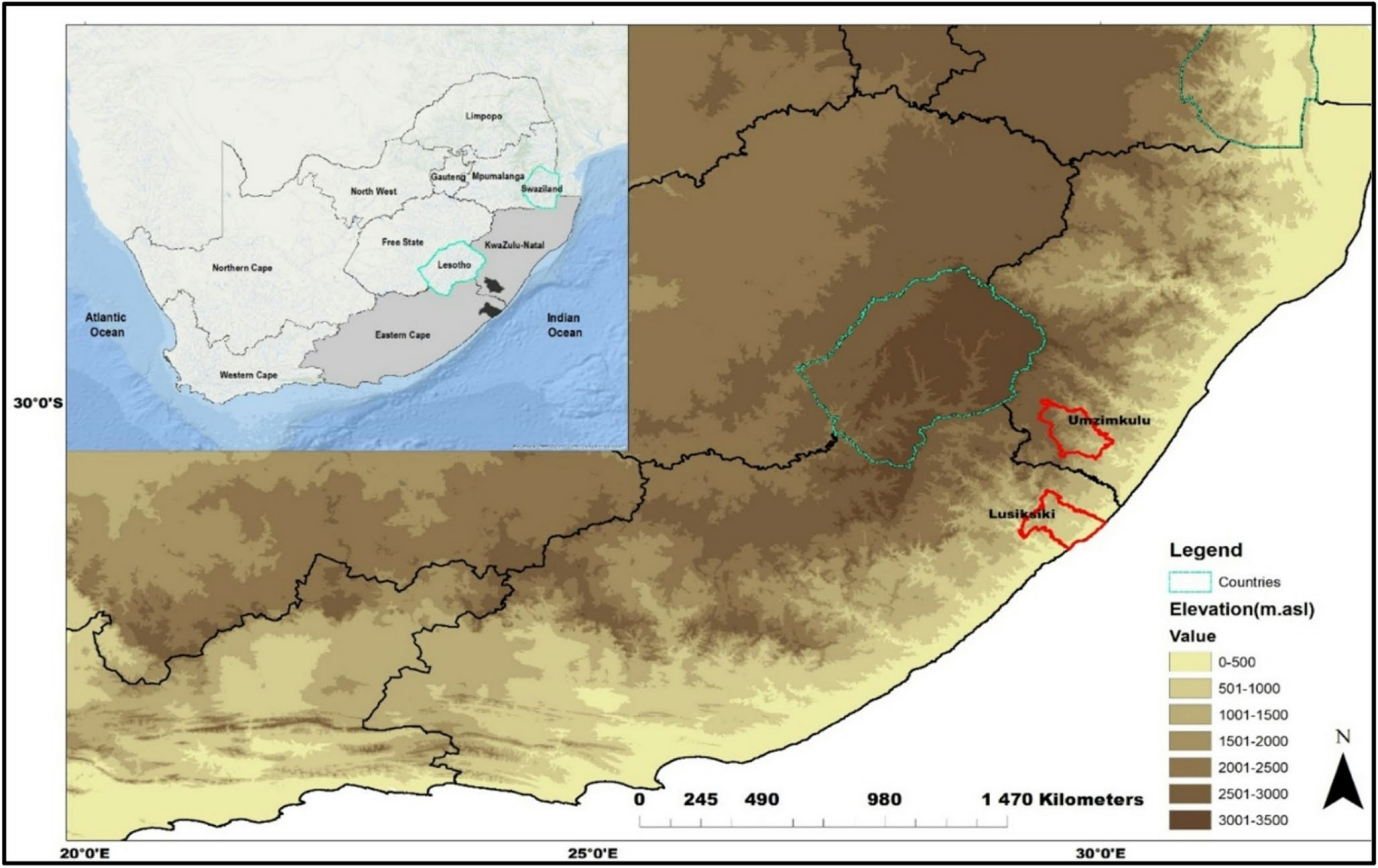
Location of Umzimkulu and Lusikiski within the KwaZulu-Natal and Eastern Cape Provinces, South Africa

The study was conducted in two South African rural areas; Umzimkulu in KwaZulu-Natal Province and Lusikisiki in the Eastern Cape Province (Fig. 1). Despite their locations across two Provinces, they are separated by only 100 km in distance (Fig. 1). Both are indigenous rural communities who are actively involved in, and rely on, small-scale subsistence agriculture. The most recent data, compiled in 2025, indicates that the most common crops that are sowed in Umzimkulu and Lusikisiki are potatoes, cabbage, and spinach (Jongisa 2005). Corn is the main staple food and commonly sowed crop (Jongisa 2005). Most community members also practice poultry farming, and more than half of the households have at least two cows and other livestock (Umzimkulu Local and Municipality 2014). The most recent Umzimkulu Integrated Development Plan (2023/2024) indicates the continued predominance of subsistence agriculture within this indigenous community, although acknowledges that there is greater scope for this than is currently being utilised. During fieldwork it was confirmed that the findings of Jongisa (2005) remained an accurate reflection of subsistence agricultural practises at the time.

Umzimkulu and Lusikisiki are both characterised by warm, humid and rainy summers with mean annual temperatures of approximately 25℃ and 800 mm rainfall that predominantly falls between September and March (Ndlovu and Demlie 2020). Winters are relatively cold and dry; mean temperatures are often below 20℃, with infrequent rainfall (Ndlovu and Demlie 2020). Agricultural activities are largely seasonal in many parts of South Africa, including Umzimkulu and Lusikisiki. Community members in Umzimkulu and Lusikisiki mostly practice rain-fed subsistence crop farming since the dissolution of the Transkei Agricultural Corporation (TRACOR) in 1995 (Jongisa 2005). Subsistence farmers predominantly rely on natural environmental conditions for productivity (Kisaka-Lwayo and Obi 2012; Rukema and Umubyeyi 2019). Over recent decades the amount and seasonality of rainfall has shifted across South Africa (Roffe et al. 2021), and temperatures have increased (Kruger and Shongwe 2004) Indigenous rural subsistence farmersin Umzimkulu and Lusikisiki have therefore had to implement adaptive strategies to ensure continued food production in the face of environmental change and changing rainfall seasonality.

### Methodology

Data for this study were collected through semi-structured interviews with indigenous subsistence farmers from Umzimkulu and Lusikisiki conducted between June and September 2019, by the first author, in the local dialect. The study involved 16 participants from Lusikisiki and 14 participants from Umzimkulu. Each participant represents a household, and often their views are informed by knowledge gained from other community members. Participants were selected purposively. The criterion for selection was developed based on the objective of the study; to identify phenological and meteorological indicators used in IKS of Umzimkulu and Lusikisiki for subsistence agricultural management and tracking seasonality (Chambers et al. 2013; Ebhuoma and Simatele 2017). The study sought community members who could provide information that represents local knowledge that has evolved through adaptive processes. Therefore, participants were selected due to their long-term residence in Umzimkulu and Lusikisiki, and their involvement in subsistence farming. We selected participants who were indigenous residents of, and practiced subsistence farming in, Umzimkulu or Lusikisiki for at least 15 years, and who are over the age of 30 at the time of data collection. This study was not focused on drawing broad generalisations about IK phenomenon or creating a statistically representative quantitative database, but rather aimed to understand IK inherent in agricultural management Umzimkulu and Lusikisiki. Therefore, the sample size was determined by data saturation, through the transcription and broad coding of each interview after it took place (Etikan et al. 2016).

Ethical clearance was obtained from the University of the Witwatersrand Human Non-Medical Research Ethics committee, protocol No: H19/04/10, before contact was initiated with interviewees, and informed consent was obtained for each individual participant. The semi-structured interviews were framed on questions pertaining to the time of year when agricultural practises take place, what prompts them, and how they gained the knowledge on the best time for these practises. The interviews were conducted at the homes of participants, and spanned approximately 45 minutes each. All interviews were audio-recorded, and transcribed verbatim and then thematically coded by the interviewer (the first author of this paper) through a deductive approach after identifying recurring information relevant to study objectives, and through consultation with the second author. Most of the themes (i.e. phenological vs. meteorology indicators, and plants vs. animal phenology) were guided by the literature prior to data collection thus facilitating comparability of the data collected. These themes are contained in the analysis conducted in this paper. Themes regarding positionality of the researchers has been published (Magaya and Fitchett 2022).

## Results

### Sample demographics

Most of the participants were officially unemployed and living on government grants. Only three respondents were retired or currently formally employed. Some respondents trade their produce, but do not engage in agriculture as a main source of income. Gender was not a variable that was considered in the analysis of these data. Nevertheless, it was noted that most of the respondents were female, which is representative of the most active demography is subsistence farming in both areas.

### Phenological awareness

Subsistence farmers in both Umzimkulu and Lusikisiki have confidence in intrinsic environmental conditions to forecast daily and seasonal weather conditions and to inform their agricultural management (Table 1). Indicators that are of particular value to both communities are phenological events, including flowering and leafing of plants, and the appearance of animals, insects and birds, meteorological conditions including cloud types and cloud cover, rainfall onset and frequency, wind direction, and astronomical observation of the sun, moon and stars (Table 1). There are no formal records of these indicators but the descriptions of phenophases by subsistence farmers from Umzimkulu and Lusikisiki indicate that both of these indigenous communities have a rich repertoire of information that contributes to agricultural management and tracking seasonality (Table 1). Residents also reflected on sensory perceptions that are indicative of changing weather conditions (Table 1).

**Table 1:** Indicators for seasonal change and daily weather used by community members in Lusikisiki and Umzimkulu

			Seasonal change	Daily weather	Respondents’ interpretations
Lusikisiki	Plants	Peach Trees	**X**		Flowering indicates spring onset. Heavy flowering indicates a good rainy season.
		Unopiyo	**X**		When this plant emerges and flowers, it is the beginning of spring.
		Grass	**X**		When the grass turns green again, this indicates spring onset
	Animals	Uvethe		**X**	Croaking of this frog indicates it will drizzle or there will be a gentle rain
		Iselesele		**X**	Croaking of this frog indicates heavy rainfall
		Goats		**X**	When goats bleat and run home, this indicates oncoming rainfall
		Southern Hornbill		**X**	The humming of this bird indicates rainfall in a few hours
Umzimkulu	Plants	Amagosi	**X**		When this plant emerges, it is spring
		Peach trees	**X**		When they flower it is spring
		Chinaberry	**X**		When chinaberry tree flowers it indicates spring onset.
		Udwabaza	**X**		The emergence of this edible wild plant indicates spring onset.
		Ucadolo	**X**		The emergence of this plant indicates spring onset.
		Mushrooms		**X**	Abundant mushrooms in certain parts of mountains indicates oncoming rainfall and thunderstorm
		Grass	**X**		When it turns green, it indicates spring onset
	Animals	Southern Hornbill		**X**	When it hums, it will rain in a few hours.
		Phezikomkhono	**X**		When they hum, it indicates approaching spring onset.
		Intlwabusuku		**X**	When they emerge it will rain or continues to rain.
		Swallows	**X**	**X**	The arrival of swallows indicates spring onset. When a flock of swallows plays in the sky it indicates rainfall, in a few days.
		Pigs		**X**	Excessive grunting of pigs indicates near rainfall.
		Isiphephane	**X**		When these birds arrive, this indicates spring onset.
Both Comunities	Meteorology	Warm winds from the east		**X**	Indicates oncoming gentle persistent rain.
		Dark clouds from the southeast (Cumulonimbus)		**X**	Indicates imminent Hailstorm and intense rainfall.
		Reddish clouds from the North		**X**	This indicates oncoming or occurs concurrently with windy conditions
		Rain clouds (nimbus clouds)		**X**	When they prevail, they indicate rainfall for that day
		Fog in the morning		**X**	Sunny conditions and high temperatures during the day.
		Rainfall	**X**		Early rainfall onset indicates a good rainfall season/ growing season
	Astronomical	Five stars aligning in South East	**X**		Indicates spring onset
		Quarter moon facing up		**X**	Indicates rainfall
		Quarter moon facing down		**X**	Indicates that rainfall is coming in a few days
		Moon with a hallo		**X**	Means that there will be windy conditions the following day or during the night
		Half-moon during the day on a clear sky		**X**	This indicates oncoming windy conditions, in a few hours. In some instances, this occurs concurrently with the windy conditions.
	Sensory	Smelling mud		**X**	Indicates oncoming rainfall, in a few hours of days
		Painful athlete’s foot		**X**	When it is raining this indicates that it will be sunny conditions for the following day and vice versa.

Respondents were aware of floral phenological indicators because of the importance of the produce that results from successful flowering of crops, and due to their consistent close spatial proximity to the plants. Animal behaviours were noted because of their seasonal emergence and their interactions with established plants and livestock. For example, respondents from Umzimkulu noted that the arrival of a flock of pied crows within seven days of sowing corn indicates that the emergence stage of corn has initiated because pied crows eat corn at this stage. Peach tree flowering is one of the most common bio-indicators used to signal spring onset. Respondents alluded that peach tree flowering is notable because peaches are a tasty and abundant fruit in the areas, and thus spring onset is significant in farming calendars. Spring onset is the most important seasonal transition in the study areas because it indicates the start of the sowing season for annual crops. Respondents from both study areas demonstrated an awareness of the relationship between meteorological conditions, phenological events (such as those listed in Table 1), and indicated that they used these in combination with the observation of astronomical cues to inform seasonality tracking and weather forecasting, thus promoting adaptation to shifting weather and climatic conditions.

As was anticipated, respondents stated that rainfall is essential for their farming activities. Therefore, are range of indicators are used to forecast the start of the rainfall season in relation to spring onset (Table 1). In both Umzimkulu and Lusikisiki meteorological indicators are predominantly used for short-term weather forecasts, such as predicting hailstorms, thus promoting short-term risk reduction decisions concerning safety and rainwater harvesting (Table 1). Meteorological indicators such as rainfall occurrence and frequency during spring are also used to make decisions relating to the commencement and type of sowing practises in both communities. Phenological indicators are important for tracking seasonal change and seasonal weather forecasting and are more significant in growing season preparedness.

A farmer from Umzimkulu explained:

*“Udwabaza (Amaranthus thunbergia*,* a wild vegetable) and ucadolo (Bidens pilosa*,* a wild vegetable) show up before we sow in our gardens*,* but they show that now we can sow.” (NMZ 1.2B)*.

Another respondent stated:

*“A year that will have enough rainfall*,* we know by…around August*,* sometimes we see from how the plants bloom*,* around August plants start blooming*,* so when they just shoot and bloom too much then we know that there will be a lot of rainfall that year. (NMZ 1.4)*

One participant explained:

*“Spring is the right time to plant*,* everything becomes alive in spring. Right now*,* we can sow*,* you see my neighbour there has some crops and even though she waters them*,* you can still see that they are winter plants. Once spring begins her garden will be more beautiful and her plants will thrive.” (NMZ 1.3A)*.

Other respondents explained that spring onset marks the timing for the onset of sowing, but the precise date for sowing commencement is determined by rainfall onset within the spring season. Sowing commencement after rainfall onset is important, and mostly prescribed for the annual staple crop, corn, because other crops such as potatoes are commonly planted in dry soil in anticipation of oncoming rainfall due to their perceived resilience to dry conditions.

The beginning of spring is marked by the flowering of plants, emergence of certain weeds (i.e., *Biden pilosa)*, and the arrival of migratory birds, including swallows. Respondents stated that, generally, the arrival of many birds after winter indicates spring season onset. However, there are three specific types of birds that are commonly indicated in seasonal and daily weather forecasting of the study areas: Phezukomkhono, barn swallows, and southern ground hornbill (Table 1).

### The reliability of IKS-informed weather forecasting

Respondents from both communities agreed that the reliability of both biological and meteorological indicators has changed, coupled changes in distribution and abundance of fauna and flora indicator species. Respondents who spoke to this, attribute these changes to environmental degradation and climate change in the study areas. One indigenous subsistence farmer, who is also a traditional healer, from Lusikisiki lamented:

*“You see my child; the way people live have changed a lot. The way we treat the environment is no longer the same. In my view that is the reason why our land is like this.” (EC 1.2)*.

This respondent further indicated that environmental changes not only reduce wild vegetable reserves, but also limit availability and access to important medicinal plants and herbs that are now mainly found in the forests. Several respondents from Umzimkulu remarked on the delay and scarcity of colourful Christmas birds that used to arrive around October and now emerge towards the end of November. Respondents indicated that this change occurred within the last decade. In Lusikisiki, respondents reflected that the plants unopiyo and unoncwembu have disappeared. These plants were important indicators for sowing onset on floodplains in the forest. Indigenous subsistence farmers in Lusikisiki used to sow in Umsikaba river flood plains earlier than at their home gardens to ensure food availability for Christmas. Although community members in this area no longer sow in Umsikaba river flood plains, the disappearance of these indicator plants demonstrates the threats of environmental change to IKS, particularly where indicator species can no longer be used. Respondents from both communities also noted the disappearance of butterflies which used to mark the onset of spring, which they attribute this to the use of chemicals such as fertilisers, pesticides, and herbicides. One participant explained:

*“We used to have butterflies*,* the yellow ones with little black spots on the wings and then the brown ones with black and white spots in the wings. They used to arrive around mid-September; I think the presence of too many chemicals in the air is the reason they disappeared.” (NMZ 2.2)*.

For short-term weather indicators, the southern ground hornbill is identified by a number of respondents as the indicator that previously had the highest accuracy for indicating oncoming rainfall, but which has predominantly lost reliability in both communities. At the beginning of an interview, one participant said:

*“You are going to make me lie because some of the most important things we relied on as rainfall indicators are not as reliable as they used to. Hearing a southern ground hornbill humming used to be a 100% indicator of rain coming in a few hours*,* but now we can hear them for a week and only receive rain in a week or two after. Although sometimes they still indicate imminent rain*,* it is not like they used to. Times have changes” (NMZ 1.2 A)*.

Although the abundance of some indicator species of seasonal variability is decreasing, some of the available indicators are still perceived to be reliable in forecasting seasonal and daily weather, and informing agricultural practise. Community members reiterated that the arrival of swallows and peach flowering remain reliable indicators for seasonal transitions.

While the reliability of plant and animal phenology for short-term weather forecasting is perceived by respondents to have decreased, meteorological indicators for short-term forecasts are argued to still have value

*Fog is an indicator that it is going to be a very hot and sunny day in summer*,* fog*,* and dew in the morning*,* meaning it is going to be very hot during the day. Even if you did not hear anything from the weatherman*,* you just know*,* and truly around 9 am it will start*,* the sun will come out. (NMZ 1.3)*

On the other hand, the reliability of medium-term meteorological indicators has decreased. Respondents from both areas mentioned that early rainfall onset in spring previously indicated a large probability of a good rainfall season, but rainfall seasonality has become more erratic, and rainfall frequency within the season has decreased, making this indicator less reliable. These changes in the reliability of both phenological and meteorological indicators indicate the considerable impacts of climate change on the natural environment, agricultural productivity and IKS-informed adaptation methods.

### Preferred weather forecasting methods

Despite the aforementioned increasingly challenges with the reliability of meteorological and phenological cues for weather forecasting and seasonality tracking, respondents still largely rely on IKS to inform the decisions that are made regarding agricultural practises. Respondents indicated a lack of trust in the seasonal weather forecasts provided by meteorologists, as it often lacks contextual relevance and has too coarse a spatial resolution. One participant in Lusikisiki explained:

*“They [meteorologists] try because we know that when they say it will rain countrywide then we will also get some water. However*,* they do not have direct information specific to our area*,* but when you hear the frog you know that it will rain here even if it is not today*,* but it will be here.” (EC 1.4)*.

Moreover, respondents from both study areas indicated that they struggle to interpret weather forecasts. One participant in Lusikisiki stated:

*“These weathermen said that we will receive 80% rainfall but as you can see outside*,* it is just drizzling*,* and that is just 30%. They are not reliable.” (EC 2.1)*.

Therefore, although the reliability of both phenological and meteorological indicators is changing, the high spatial resolution offered by IKS and the local familiarity of IKS compared to the meteorological weather forecasts promotes its continued use in Umzimkulu and Lusikisiki. This underscores the importance of IKS in understanding phenological observations in the Global South, as it is unlikely to be unique to these two communities.

## Discussion

Observing changes in the natural environment throughout seasons is common across a wide range of communities across the world, and predates the availability of contemporary weather forecasts in many rural communities across the world (Armatas et al. 2016; Basdew et al. 2017; Clapcott et al. 2025). These observations, and the interpretations that derive from them, vary between communities, between areas within a community, and between homes in an area (Eakin 1999; Dlamini and Ocholla 2018; Egeru 2012; Goduka 2012). Respondents from Umzimkulu and Lusikiski forecast both daily weather and seasonal conditions through observing several local environment variables including the onset of rainfall in summer, the appearance of birds, and rainfall frequency at the beginning of spring. Rainfall and overall climatic viability are a serious consideration in agriculture for many Sub-Saharan African countries because rain-fed subsistence farming is an important contributor to food availability and access (Calzadilla et al. 2013; Davis 2011; Ajani et al. 2013; Shisanya and Mafongoya 2016). Therefore, many phenological and meteorological indicators in Umzimkulu and Lusikisiki are used to forecast rainfall patterns (Table 1). This information is used to frame, quantify and understand time (Chisholm-Hatfield et al. 2018; Chambers et al. 2021; Bastian and Bayliss Hawitt 2023), and specifically for these communities, to mark sowing season onset, sowing dates within the sowing season, and preparing social and financial capital for a growing season. Indigenous subsistence farmers interviewed from Umzimkulu and Lusikisiki mostly have limited financial capital and cannot afford significant agricultural losses that may arise due to inaccurate weather forecasts, or a misinterpretation of their content, as occurred for the Delta state of Nigeria in 2012 (Ebhuoma and Simatele 2019). In sub-Saharan Africa, the use of IKS has been seen as a key strategy for climate change adaptation (Ajani et al. 2013).

The availability of accurate, timely, spatially-specific and context-efficient weather forecasts and warning systems can reduce the risk of climate stresses to farming activities and promote food production (Kgakatsi and Rautenbach 2014; Plotz et al. 2017; Chambers et al. 2021). Phenologically-informed IKS has been valuable in providing spatial specific information with considerable temporal depth to many rural communities through oral histories (Eakin 1999; Chambers et al. 2013). Bird migration patterns are one of the most important phenological indicators that are commonly used in IKS. Birds’ departure and arrival dates have been used in Mexico (Eakin 1999), Uganda (Orlove et al. 2010) and in northern KwaZulu-Natal (Rukema and Umubyeyi 2019) to forecast short and medium-term weather conditions. In Umzimkulu and Lusikisiki, the arrival of migratory birds such as swallows is an important indicator of spring onset. A global statistically significant shift in the timing of bird migration due to climate change has been established (Gordo 2007). Community members in Umzimkulu and Lusikisiki have noted shifts in the arrival dates of some bird species, and a marked decrease in abundance and disappearance of others. These changes in bird migration patterns and abundance due to environmental change will affect the reliability of phenologically-derived IKS cues for agricultural activities and threaten agricultural productivity in many rural areas (Tomotani et al. 2018 Vähätalo et al. 2004). However, continued in situ phenological indicator observations promote ecosystem monitoring and extensive understanding of seasonal events and may continue to facilitate pragmatic adaptation to environmental change. Furthermore, IKS can contribute to the understanding of different species’ phenology and the climate drivers that influence shifts in specific areas (Fitchett and Ebhuoma 2018).

Community members in Umzimkulu and Lusikisiki recognise that the reliability of some phenological indicators has changed considerably, but reflect that the spatial-specificity of IKS cues outweighs the quantified but less contextually specific forecasts provided by meteorologists. In the context of phenological shifts and extirpation of indicator species, these communities may benefit in the long term from the integration of knowledge systems, and greater engagement from meteroologists on the communication and translation of forecast information and nomenclature. Merging IKS-derived cues for weather and seasonality with contemporary weather forecasts to provide forecasts that have high spatial resolution and contextually relevant for agricultural rural areas is argued to be a valuable component for improving the uptake of weather forecasts (Chambers et al. 2017, 2021; Ebhuoma and Simatele 2017). Furthermore, there is already a degree of consistency in IKS-informed weather forecasting indicators between different communities across South Africa, which further promotes the potential to synthesise IKS indicators with traditional weather forecasting. For example, peach tree flowering is also used to indicate spring season onset in uMgungundlovu and Msinga; rural areas in KwaZulu-Natal, and by other agricultural rural communities in the Free State Province, South Africa (Basdew et al. 2017; Rukema and Umubyeyi 2019; Zuma-Netshiukhwi et al. 2013).

The formal documentation of IK with the development of medium to long-term phenological datasets for the country could facilitate the integration of knowledge systems (Chambers et al. 2017; Plotz et al. 2017), which is paramount in addressing climate change impacts in ways that are beneficial to both urban and rural regions (Bisong and Andrew-Essein 2010). However, there are controversies regarding the protection of the IK and sustaining its integrity throughout the process of documentation and dissemination (Hatfield 2009; Chambers et al. 2017; Moahi 2007). Chambers et al. (2017) highlight issues relating to appropriate means to store and manage the information that will be collected from locals communities, removing their ‘ownership’ of knowledge, a concern echoed in South Africa (Dlamini and Ocholla 2018; Moahi 2007). Chambers et al. (2017) argue that when IK databases are developed and managed by other parties other than the knowledge holders, the protection of knowledge holders’ rights, cultural and intellectual property sensitivities need to be considered more attentively. Therefore, although documenting and storing IK is recommended, the obscured intellectual property rights for community-owned knowledge may render IKS vulnerable to exploitation (Chambers et al. 2017; Tong 2017). Nonetheless, continued research, where similarly respondents provide informed consent and where care is taken to not be extractivist in nature, that documents IKS biological indicators and integration of the knowledge systems with in-depth ethical consideration for indigenous communities will be a significant contribution to climate change adaptation.

## Conclusion

Due to their long-term residency in Umzimkulu and Lusikisiki, respondents of this study represent cumulative knowledge of weather patterns and changes in the phenology of indicator species. When rural, indigenous subsistence agriculture dependent communities detect, understand, and respond to environmental change, they can effectively limit the risks and adapt to the impacts of climate change (Lauer and Aswani 2010; Clapcott et al. 2025). Nevertheless, due to a lack of resources and limited trust in national meteorological services by many small-scale indigenous rural farmers based on misalignment between forecasts and weather events, including the participants of this study, the current and projected changes in species phenology pose a significant threat to adaptation strategies. Therefore, in addition to undermining adaptation options, changes in phenology and disappearance of indicator species may lead to permanent loss of significant knowledge linked to the culture of some populations. For future research and praxis, more deliberate mixed-methods approaches to forecasting, and knowledge system integration approaches can potentially be used to enhance current adaptive strategies, may provide alternate options for indigenous rural communities to respond quickly to climate variability and change, while recognising and respecting the value in their TPK.

## Data Availability

Due to the requirements of confidentiality through the ethics clearance granted, data are not publically available.
